# *Emergomyces orientalis* Emergomycosis Diagnosed by Metagenomic Next-Generation Sequencing

**DOI:** 10.3201/eid2710.210769

**Published:** 2021-10

**Authors:** Da He, Min Quan, Hongyan Zhong, Zhixing Chen, Xioahui Wang, Fang He, Junyan Qu, Taoyou Zhou, Xiaoju Lv, Zhiyong Zong

**Affiliations:** Center for Infectious Diseases, West China Hospital of Sichuan University, Chengdu, China (D. He, M. Quan, X. Wang, F. He, J. Qu, T. Zhou, X. Lv, Z. Zong);; Hospital of Chengdu Office of People's Government of Tibetan Autonomous Region, Chengdu (H. Zhong);; West China Hospital of Sichuan University, Chengdu (Z. Chen);; Center for Infectious Diseases, Yaan People’s Hospital, Yaan, China (X. Wang)

**Keywords:** China, disseminated emergomycosis, Emergomyces, Emergomyces orientalis, fungi, immunocompromised, metagenomic next-generation sequencing, respiratory infections, Tibet, transplant patients

## Abstract

*Emergomyces* is a newly described dimorphic fungus genus; it may cause fatal infections in immunocompromised patients, but diagnosis is often delayed. We report a case of disseminated emergomycosis caused by the novel species *Emergomyces orientalis* in a kidney transplant recipient from Tibet. Infection was diagnosed early by metagenomic next-generation sequencing.

Emergomycosis (formerly called emmonsiosis) is an emerging dimorphic fungal disease, usually caused by *Emergomyces pasteurianus* or *Es. africanus*, usually disseminated and commonly identified and fatal in immunocompromised patients, especially HIV-positive patients from South Africa (*1*,*2*). Diagnosis of emergomycosis is often delayed, and best clinical practices for diagnosing and treating organ transplant recipients are lacking. Five species with different geographic distributions have been described: *Es. pasteurianus*, *Es. africanus*, *Es. canadensis*, *Es. europaeus*, and *Es. orientalis*. Globally, the only case of *Es. orientalis* infection, reported in China in 2017, was initially misdiagnosed as disseminated cryptococcosis (*3*). We report another case of *Es. orientalis* infection involving lung and soft tissue damage that was diagnosed early and accurately and treated precisely.

A 41-year-old man from Tibet who had received a kidney transplant 6 years earlier was admitted to a hospital with a 1-month history of progressive right lower chest pain and mild cough with a small amount of sputum. He was taking tacrolimus, mycophenolate mofetil, and prednisone. He was a herder caring for sheep, horses, and dogs. We noted reduced breath sounds in his lower right lung; chest computed tomography images indicated pneumonia (Figure, panel A). A bronchoalveolar lavage fluid smear revealed yeast-like fungi on both Gram staining and Grocott-Gomori methenamine silver staining (Figure, panel B). Because pulmonary cryptococcosis was suspected, fluconazole (400 mg 1×/d) was initiated. Results of a cryptococcal antigen lateral flow immunoassay (IMMY, https://www.immy.com) was negative, but a Platelia *Aspergillus* antigen immunoenzymatic sandwich microplate assay (Bio-Rad, https://www.bio-rad.com) resulted in an unexpectedly high level (6.42 [reference 0.00–0.49] signal:cutoff ratio). After 1 week of ineffective empirically prescribed treatment, we had a lung biopsy performed. Electron microscopy revealed yeast cells in a unique form, measuring ≈3 μm, scattered in necrotizing granulomas (Figure, panel C). Metagenomic next-generation sequencing (mNGS) of fresh tissue indicated *Es. orientalis* (sequence reads 143;, Illumina NextSeq 550 platform, https://www.illumina.com; Appendix Figure 1). We initiated oral itraconazole (200 mg 2×/d) immediately and decreased tacrolimus dosage according to its plasma concentration. Finally, we isolated the pure *Es. orientalis* strain (Figure, panel D). Specific secondary, α-shaped conidiophores clearly indicated *Emergomyces* (Figure, panel E). *Es. orientalis* was confirmed by PCR amplification targeting the rDNA internal transcribed spacer region followed by BLAST sequence comparison (https://blast.ncbi.nlm.nih.gov/Blast.cgi; GenBank accession no. NR_148064.1; coverage 96%, identity 99.33%) (Appendix Figure 2).

**Figure:**
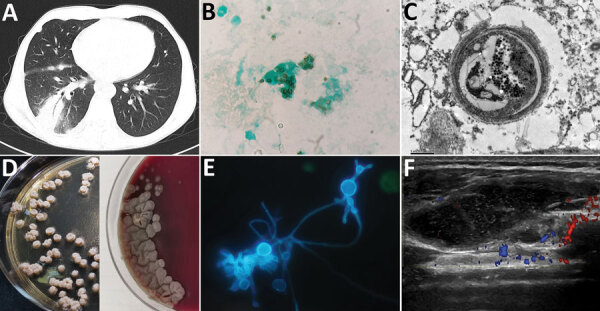
*Emergomyces orientalis* infection in a kidney transplant patient from Tibet. A) Pulmonary consolidation with the air bronchogram sign shown on a computed tomography scan. B) Microbes stained with Grocott-Gomori's methenamine silver in the bronchoalveolar lavage fluid sample (original magnification ×1,000). C) Pathological image of 1 yeast cell shown by electron microscopy in a necrotizing granuloma from paraffin-embedded pulmonary tissue (original magnification ×16,000). D) Tiny, slightly raised white colonies on Sabouraud agar on day 20 at 25°C (left) and grayish yellow furrowed colonies on blood agar on day 30 at 35°C (right) isolated from bronchoalveolar lavage fluid samples. E) Specific secondary α-shaped conidiophore shown with fluorescent calcium staining (original magnification ×1,000). F) Ultrasound revealed a soft tissue abscess in the patient’s right subcostalis.

During treatment, the patient had intermittent mild fever and an acne-like rash on his chin, and a small new pulmonary lesion developed in the right upper lobe. Repeated blood cultures were all negative. We prescribed oral posaconazole (400 mg 2×/d) after determining a MIC of 0.008 μg/mL (Appendix Table). Later, the lung lesions partially resolved, but we found a painful soft tissue abscess (55 × 15 × 30 mm) on the right side of his waist (Figure, panel F) from which we drained purulent grayish-green fluid. We again cultured *Es. orientalis*. Therefore, we added flucytosine (1,000 mg 3×/d) and withdrew tacrolimus and mycophenolate mofetil for 1 month. After 6 months of recurrent hospitalization, we discharged the patient with a diagnosis of disseminated emergomycosis. Six months after discharge, he remained stable. We found no similarly infected or epidemiologically linked person or animal.

Previously, a retrospective study from southern Africa assessed 54 patients with disseminated emergomycosis, of whom 94% were co-infected with HIV; 96% had skin involvement, 88% had lung involvement, 44% received an incorrect diagnosis, and 48% died (*4*). In this case, we initially identified *Es. orientalis* infection using mNGS, a 1-step, culture-independent method for detecting all pathogens from 1 specimen (*5*). Although research validating mNGS assays in clinical practice is very limited, challenging cases diagnosed by mNGS have been published and expert consensus has begun to recommend mNGS for diagnosing challenging cases in immunocompromised patients (*6*,*7*). Therefore, we recommend using mNGS to diagnose challenging emergomycosis cases.

This case showed that treatment with posaconazole combined with flucytosine is effective in organ transplant recipients with disseminated emergomycosis caused by *Es. orientalis.* Although amphotericin B deoxycholate is more effective than triazoles for improving emergomycosis survival rate (71% vs. 33%) (*4*), we could not prescribe it for our patient because of nephrotoxicity. Similar to the earlier reported case of *Es. orientalis* infection, in which type 2 diabetes was the only identified cause of immunodeficiency (*3*), fluconazole was ineffective in vivo in our patient. Previously, 3 cases in China of *Es. pasteurianus* (formerly *Emmonsia pasteuriana*) infection with or without renal transplantation have also been reported (*8*–*10*). 

Further research is needed to determine whether kidney transplantation is associated with *Es. orientalis* infection and risk for emergomycosis. In conclusion, clinicians need to become more aware of emergomycosis because of its common misdiagnosis and high death rate. 

Appendix Additional information about emergomycosis caused by *Emergomyces orientalis* and diagnosed by metagenomic next-generation sequencing.
