# Correction: Vol. 27, No. 10

**DOI:** 10.3201/eid2712.C12712

**Published:** 2021-12

**Keywords:** errata, erratum, correction

The name of author Xiaohui Wang was misspelled in *Emergomyces orientalis* Emergomycosis Diagnosed by Metagenomic Next-Generation Sequencing (D. He et al.). The article has been corrected online (https://wwwnc.cdc.gov/eid/article/27/10/21-0769_article).

