# Supplementary material for: Emergomyces orientalis Emergomycosis Diagnosed by Metagenomic Next-Generation Sequencing
Source: Emerg Infect Dis. 2021 Oct;27(10):2740–2. doi: 10.3201/eid2710.210769 (PMC8462323; doi:10.3201/eid2710.210769)
Supplement: Appendix — Additional information about emergomycosis caused by Emergomyces orientalis and diagnosed by metagenomic next-generation sequencing. [file 21-0769-Techapp-s1.pdf]

Article DOI: https://doi.org/10.3201/eid2710.210769

Emergomyces orientalis Emergomycosis Diagnosed by Metagenomic Next-Generation Sequencing Appendix

Appendix Table. Antimicrobial susceptibility test results*

Antimicrobials	MIC, µg/mL
amphotericin B	0.2
5-fluorocytosine	0.06
fluconazole	1
itraconazole	0.015
voriconazole	0.015
posaconazole	0.008
anidulafungin	8
caspofungin	1
micafungin	8

*Performed in the yeast phase using the fungal broth microdilution method.

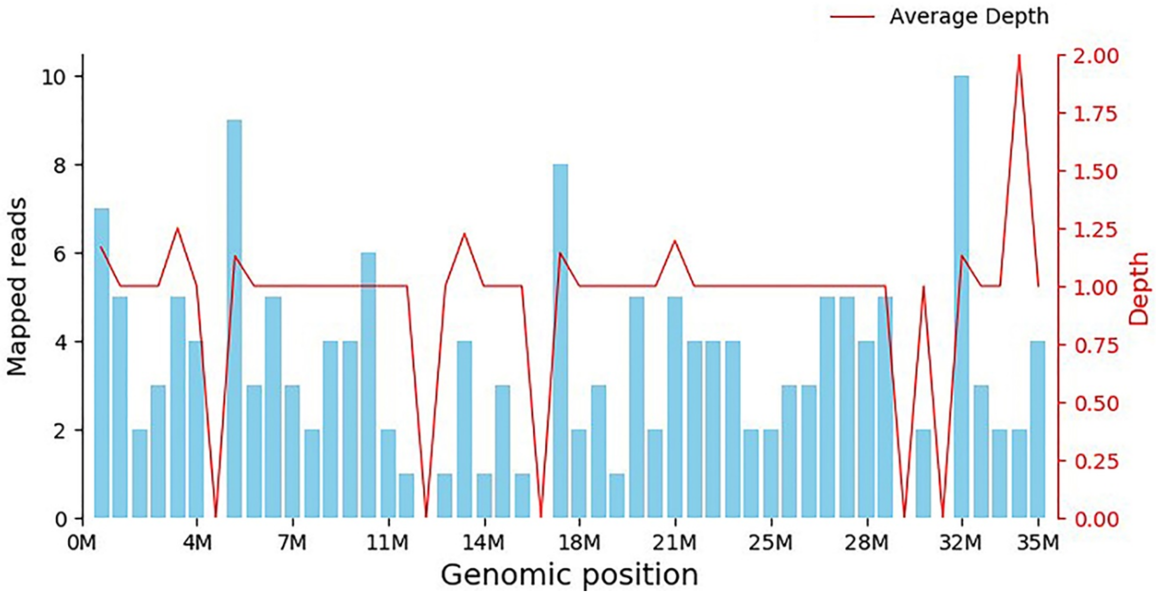

Appendix Figure 1. *Emergomyces orientalis* coverage map. The identified reads were mapped to the *Es. orientalis* reference genome (GenBank accession no. GCA_002110485.1). The abscissa is the genome position of *Es. orientalis* reference genome. The left-side scale shows the number of matched mNGS sequences in the alignment. The right-side scale shows the sequencing depth (i.e., the number of times the base pair site has been sequenced). The blue bars show the number of matched mNGS sequences corresponding to various positions in the genome of the fungus. The red line represents the average sequence depth distribution at different positions in the bacterial genome. M, position in the genome in millions of base pairs (x-axis scale)

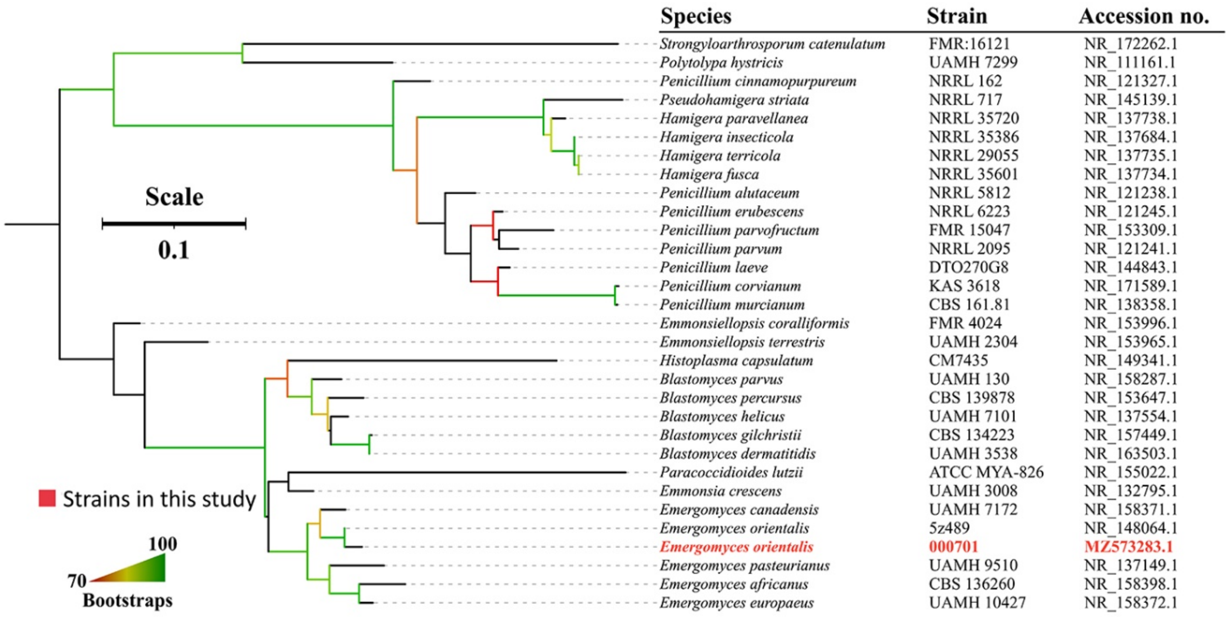

Appendix Figure 2. Phylogenetic tree of the ITS sequences amplified from the isolate in this study and sequences from reference strains in GenBank. A maximum likelihood tree was inferred using IQ-TREE under the TIM2+F+I+G4 model, chosen by the program according to the Bayesian information criterion and subjected to a 10,000-iteration bootstrap test to check the robustness. The supporting values were colored in gradients and the branch lengths were measured in terms of the number of substitutions per site. The scale bar represents number of substitutions per site.
